# Associations between social factors and school belonging among newcomer and non-newcomer youth in Sweden

**DOI:** 10.1371/journal.pone.0280244

**Published:** 2023-02-03

**Authors:** Serena McDiarmid, Fatumo Osman, Anna Sarkadi, Natalie Durbeej

**Affiliations:** 1 Department of Psychology, University of Waterloo, Waterloo, Canada; 2 Department of Public Health and Caring Sciences, Uppsala University, Uppsala, Sweden; 3 School of Health and Welfare, Dalarna University, Falun, Sweden; Chiang Mai University, THAILAND

## Abstract

Feeling a sense of belonging at school is associated with important positive outcomes for youth and requires youth to engage in positive social relationships. Yet there is a limited understanding of the social factors most associated with youths’ school belonging and limited evidence about whether correlates of school belonging vary for marginalized groups like newcomers compared to majority groups. Sweden provides an important context for investigation of these issues because, over the past two decades, the country has experienced an influx of asylum seekers and educational reforms that have altered the composition and functioning of Swedish secondary schools. This study addresses these gaps by (1) investigating which of eight social factors are associated with school belonging among diverse Swedish youth, and (2) examining whether newcomer status moderates the relationship between social factors and school belonging. Hierarchical regression and moderation analyses were used to analyze data from 14 to 19 year-old (*n* = 233) newcomers and non-newcomers in Sweden. An exploratory factor analysis revealed that the school belonging measure contained two factors: positive perceptions and negative perceptions (reverse coded). For both, stronger school belonging was associated with lower perceived ethnic discrimination. Positive perceptions of school belonging were also associated with more prosocial behaviours and lower emotional problems. Negative perceptions of school belonging were associated with more peer problems. Notably, quantity and quality of peer relationships were not associated with school belonging. There was no consistent evidence of newcomer status moderating the relationship between social factors and school belonging. These results highlight factors associated with school belonging which are modifiable and amenable to intervention or impact by policy—ethnic discrimination, prosocial behaviour, and emotional and peer problems. The absence of moderation by newcomer status suggests that school belonging interventions or related policies are likely to affect newcomer and non-newcomer students similarly.

## Introduction

### Background

Belonging is a fundamental human need [1–3] and may be especially important during adolescence as youth navigate identity formation outside their family and an increasing importance is placed on non-family relationships [4, 5]. According to the belongingness hypothesis, feeling a sense of belonging requires frequent, positive personal contact with others and perceiving one’s bonds with others to be stable [3]. Social relationships therefore play an important role in feeling belonging, and schools provide an important social context in which youth may form social relationships and therefore establish a sense of belonging [6–9].

A growing body of research has investigated belonging in schools. School belonging is frequently described as the “…extent to which students feel personally accepted, respected, included, and supported by others in the school environment” [10, p 80]. In many studies, school belonging has been used synonymously with school attachment, school connectedness, school engagement, school identification, school relatedness, or a sense of school community, among other terms [11–13], while being contrasted with concepts like school achievement [14].

School belonging plays an important role in youths’ lives. Many have argued that a sense of belonging is necessary for learning and academic success [7, 15, 16] and school belonging has been positively associated with academic success in prior work [12, 14, 17, 18]. Other important variables like happiness and wellbeing [19–21] and mental health [22–25] are also associated with school belonging. A recent meta-analysis of 82 studies affirmed the importance of school belonging by demonstrating positive correlations between school belonging and academic achievement, academic engagement and effort, and positive self-perceptions as well as a negative correlation between school belonging and school leaving, even when accounting for possible publication biases [14].

### School belonging in the Swedish context

Over the past two decades, school belonging levels have declined in Sweden [13], as they have internationally [18]. Since 2012, this decline in Sweden has been greater for foreign-born youth than native Swedes [13]. During the same time period, Sweden has experienced an influx of displaced persons and asylum seekers, many of whom are school-aged [26], and a reform of the educational system resulting in a greater focus on evaluation and performance [13, 27–29]. Swedish law dictates that newcomer youth may enter the school system immediately upon claiming asylum in the country, which has resulted in a high proportions of refugee youth in the Swedish school system [30–32]. These changes in migration patterns and educational policy have altered both the functioning and composition of Swedish secondary schools, providing a unique context in which to study the social factors predicting school belonging among newcomer and non-newcomer youth.

### Social correlates of school belonging

Because belonging derives from frequent, positive contact and stable bonds with others, social factors can be expected to be associated with school belonging. Here we review the literature on several social correlates of school belonging, many of which are proximal to the individual. Nonetheless, there are other factors distal to the individual, such as educational policy, geography or cultural norms, that may act as correlates and these factors may also influence the development or expression of the social correlates described here [33–35].

One social factor hypothesized as a correlate of school belonging is the quality of youth’s peer relationships. Hamm & Faircloth [36] have argued that positive peer relationships serve as “a secure base and buffer”, helping youth to successfully navigate secondary school challenges and feel a sense of school belonging. Osterman [7] has similarly argued that peer acceptance and friendships contribute to a students’ sense of belonging to the school community. These arguments have been borne out by research showing associations between school belonging and the perceived quality of peer relationships [37, 38] and acceptance and social support among peers [39–42]. This pattern of results has also been observed among newcomer youth; in Fazel’s [6] interviews with adolescent and young adult refugees resettled in the UK, participants reported feeling peers were an especially important source of acceptance in secondary school, even more than teachers.

Less is known about whether youths’ quantity of peer relationships predicts school belonging and no previous study, to our knowledge, has explored this association. A positive association may be expected, given the theoretical relationship between friendships and school belonging. However, Galatzer-Levy and colleagues’ [43] investigation of coping among youth in college revealed that the quality and not the quantity of social relationships predicted successful adaptation and a similar pattern may emerge for youths’ school belonging.

School’s social environment, such as whether the social context is emotionally supportive, positive, and non-discriminatory, also affects youths’ experiences of school belonging [33]. Perceiving discrimination within a school context is particularly associated with lower feelings of belonging at school [44, 45] and the effects of discrimination are stronger for some ethnic-racial groups [46]. Considering belonging in general, a large Canadian study also found that, while discrimination is negatively associated with belonging for individuals in general, the association is stronger among racialized minorities [47]. These findings suggests that, for youth who are part of a racialized newcomer group, discrimination may predict school belonging more strongly.

Finally, an individual’s social functioning is likely to be associated with school belonging. In their meta-analysis of 51 studies, Allen et al. [33] examined several domains as possible correlates of school belonging. These domains included “emotional stability” which encompassed variables like depression and emotional problems, and the domain of “personal characteristics” which included variables like positive affect and prosocial goal pursuits. The domains called “emotional stability” and “positive personal characteristics” were observed to be correlates of school belonging. Other studies since have also found associations between school belonging and psychosocial functioning, including internalizing problems like depression and externalizing problems like conduct issues [23, 25, 39, 48–51], and positive relational skills like prosociality [25, 51, 52]. The direction of these associations is unclear. Prior literature in this area has been mixed with some supporting behaviour as an antecedent to school belonging [e.g., 53], some suggesting school belonging influences behaviour [e.g., 54], and others hypothesizing a reciprocal relationship [e.g., 14, 49].

Limited work has also examined social functioning and school belonging among youth with a refugee background and found a similar pattern of results [55]. Notably, rates of internalizing and externalizing problems tend to be higher among refugee newcomer youth than youth in the general population [56–60].

Age, gender and material access are also often examined in relation to school belonging. A decline in school belonging with age has been observed [40, 50, 61]. Gender differences in school belonging have also been found [33, 62, 63] though the direction is not consistent [18] and not all studies have found such differences [e.g., 15]. Finally, one’s relative economic advantage has been found to be positively associated with school belonging across almost all education systems that participated in the 2015 and 2018 Programme for International Student Assessment (PISA) studies [18, 64].

### School belonging among newcomer youth

Limited work has focussed on school belonging among newcomer youth, including those with a refugee background [e.g., 55]. Several qualitative studies have reported that refugee youth feel a lack of belonging in schools and this lack of belonging is often attributed to experiences of discrimination in school and struggles forming peer friendships [6, 65, 66]. Struggles forming peer relationships can arise for many structural reasons such as frequent relocation of refugee youth due to immigration policies, institutional cultural barriers, or a lack of mental health resources within the healthcare system for youth experiencing mental distress [9]. A large-scale study using data from the 2018 PISA found lower school belonging among youth with immigrant backgrounds compared to native youth in about a third of education systems [18]. Given the limited research in this area, Slaten and colleagues [12] have called for additional research into how school belonging may be experienced differently by youth in marginalized groups. It is therefore important to investigate social correlates of school belonging among newcomer youth and whether the relationships between school belonging and social correlates are different for this group compared to non-newcomer youth.

### Aims

While some prior research has examined correlates of school belonging, few have focused specifically on which social factors are associated with school belonging in a highly diverse sample of youth and if these associations are moderated by newcomer status. Using cross-sectional data, we aim to address these gaps in the literature by:

1. Investigating which social factors are associated with school belonging among diverse Swedish youth, and
2. Examining whether newcomer status moderates the relationship between social factors and school belonging

Addressing these aims will be beneficial to researchers looking to better define the construct of school belonging; to practitioners hoping to refine school belonging interventions, especially those focussed on asset-building [67], to make them more effective, especially for refugee youth; and to policy makers who oversee structures and policies that can shape school belonging.

## Methods

### Study design

The study was part of the RefugeesWellSchool project, a larger intervention study aiming to evaluate various school-based interventions for promoting mental health among refugee and migrant children and youths [68]. We used cross-sectional data from the first, baseline measurement.

### Participants and setting

Data were collected in nine Swedish schools located in both rural and urban areas from September 2019 to February 2020 [68]. Schools that had students in grades 7–9 and/or introduction classes for newcomers, and a high proportion of multi-ethnic students (i.e., ≥30% of registered students have a non-Swedish background) were recruited to participate through phone and email contact with municipalities and school administrators. Of 72 schools contacted, nine agreed to participate in the intervention study and thus baseline data collection.

Within participating schools, all students in introductory classes for newcomers and in traditional classes of grade 7–9 were invited to participate in data collection and no upper limit was set for the sample size. A total of 541 students attended a short information session during class time and received an informed consent document. To participate, students had to provide their informed consent and those younger than 15 also needed consent from their guardians as mandated by ethics law. In all, 302 participants (56%) agreed to participate in the survey and 280 (52%) submitted at least partial survey data and adequate consent forms.

Participating students completed the measures electronically in a quiet space during class time. Survey measures were presented using LimeSurvey [69] and were available in 26 languages. Participants were assisted in completing the survey, as needed, by the research teams or by their classroom or first-language teachers.

### Measures

#### Demographics

Youths’ demographic information including age in years and gender were collected, along with whether the youth is a newcomer to Sweden. Newcomers were defined as anyone who arrived in Sweden ≤ 6 years ago.

#### Material access

Access to material goods was measured using six items from the material-stress-items of the Daily Stressors Scale for Young Refugees [70]. The scale was developed based on the Columbia Impairment Scale [71] and Adolescents Complex Daily Stressors Scale [72] for use with unaccompanied refugee minors in Belgium. For each item, youth responded on a four-point Likert scale from 1 (*never*) to 4 (*always*) or with “*I don’t know*” to the question of how often they had experienced material deprivation in the prior month with regard to housing, food, medical care and other items. The scale is beginning to be more widely adopted [54, 73] and there is emerging evidence to show the full scale has good internal consistency [73]. For data analysis, answers were reviewed and responses of “I don’t know” were coded as missing data. An average score between 1 and 4 was then calculated for every participant who answered one or more of the items, with a higher score indicating higher access to resources. Data was considered missing for participants who didn’t answer or responded “*I don’t know*” to all items.

#### Psychological sense of school membership scale

School belonging was measured using nine items from the 18-item Psychological Sense of School Membership (PSSM) Scale [10]. The nine items used included four with the self as the referent and five with the school as the referent [74]. As discussed in the “Exploratory Factor Analysis” section below, one of the nine items was ultimately dropped due to inappropriate factor loading. Therefore, to create the variable used for analysis, the four negatively worded items were reverse coded, then the eight retained items were summed to create a total variable score between 8 and 40.

This measure was selected for several reasons: It is the most widely-used measure of school belonging [14]; it has successfully been shortened by others [19, 75, 76] and therefore is accommodating to administration time constraints; and the full measure has been shown to have strong psychometric properties including good internal reliability [see 77 for a review], strong construct validity, and moderate to high test-retest reliability [78, 79].

However, the PSSM is not without controversy. While some have found a good fit for a unidimensional factor structure [10, 80], many have demonstrated through factor analysis that the measure is multidimensional [74, 77, 81, 82] and some have suggested that, based on face validity, the PSSM may measure multiple related constructs [77]. To address the possibility of multidimensionality, we first conducted a factor analysis on the PSSM and then analyzed the data based on the two subscales identified in the factor analysis. Possible interpretation of these two dimensions is outlined in the Discussion.

#### Friendship measures

Friendship quantity was measured by self-report of the youths’ total friends (scale: 0–20) from (a) Sweden, (b) their country of origin, and (c) not from Sweden or their country of origin. Total number of friends was calculated by summing the three numbers.

Friendship quality was measured using the Multidimensional Scale of Perceived Social Support [83]. The measure was developed to assess social support, and the four items related to social support in friendships were used. The measure has shown high internal consistency among multiracial youth internationally [84–86]. Youth responded on a four-point Likert scale from 1 (*not at all*) to 4 (*a lot*), or “I don’t know”, to indicate their level of agreement with statements like, “I can count on my friends when things go wrong.”. An average score from one to four was calculated for each participant who answered one or more of the items. Data was considered missing for participants who didn’t answer or responded “I don’t know” to all items.

#### Perceived ethnic discrimination questionnaire

Perceived ethnic discrimination was measured using nine-items from the Perceived Ethnic Discrimination Questionnaire-Community Version Brief (PEDQ-CVB) including the Discrimination at Work/School and Discrimination/Stigmatization subscales [87]. The PEDQ-CVB subscales have demonstrated adequate to good internal reliability in studies with different racial/ethnic groups [87–89] and evidence suggests members of different racial groups interpret the items in a similar manner [90]. Participants responded on a four-point Likert scale from 1 (*never*) to four (*always*) or “I don’t know/I don’t want to answer” to questions about the frequency of discriminatory events such as, “Has it been hinted that you must be lazy?” and “Have others thought you couldn’t do things or handle a task?”. An average score from one to four was calculated for every participant who answered one or more of the items. A higher score indicates higher levels of perceived ethnic discrimination. Data was considered missing for participants who didn’t answer or responded “I don’t know/I don’t want to answer” to all items.

#### Strengths and difficulties questionnaire

The Strengths and Difficulties Questionnaire (SDQ) is a widely used measure of internalizing and externalizing problems [91] and is used here as a measure of youths’ social functioning. The measure consists of five subscales, each with five items: Prosocial behavior, Emotional symptoms, Conduct problems, Hyperactivity, and Peer problems. The measure has good construct and criterion validity [92] and has demonstrated good internal reliability in a similar study of adolescent students in Sweden [93] and in other studies [91, 94, 95]. However, some have found poor reliability, especially on the conduct and peer problems subscales [96].

Participants respond with the degree to which each statement on the measure applies to them on a scale of zero (*not true*) to two (*certainly true*). For analysis, necessary items are reverse coded according to the SDQ manual. The scores for each item of the subscale are then summed, creating a total score between zero and ten. A larger score on the Prosocial behaviour subscale is intended to indicate higher prosocial behaviour and stronger social functioning. Higher scores on the remaining four subscales are intended to reflect higher levels of internalizing or externalizing problems and poorer social functioning. While an overall total difficulties score can be created, we chose to include each subscale in the analysis individually.

### Ethics

The study received ethical approval from the Regional Ethical Review Board at Uppsala University (dnr: 2019–031160). All participants were informed orally and in writing that participation was voluntary, that confidentiality would be maintained, and that they could withdraw from the study at any time without explanation. All participants gave their informed consent to participate and all youth under 15 also had consent from a parent or guardian to participate.

### Statistical analyses

This study uses cross-sectional data and therefore makes no claims about the direction of association between school belonging and the social factors we investigate. School belonging is positioned as the dependent variable within this analysis because the construct is of paramount importance within the lives of youth.

Descriptive statistics including means, standard deviations, ranges, frequencies and proportions were examined and used to describe the sample and Pearson correlations were also calculated between all independent variables and the dependent variable. Cronbach’s alpha coefficients were calculated as measures of internal consistency and independent t-tests were conducted for group comparisons.

Of 280 responses with at least partial data, 233 were included in the analysis; four participants were excluded for spurious answering patterns and 43 participants were excluded for missing data. The most commonly missing variables were total friends (*n* = 24), followed by newcomer status (*n* = 12), and age (*n* = 7). The assumption of normality was met for all variables, as all had a skew < 3 and kurtosis <10. Multicollinearity was assessed by computing Pearson correlations between all independent variables. None of the correlations exceeded 0.70. The significance level was set at *p* < .05 and all analyses were computed using SPSS version 27.

A two-stage hierarchical multiple regression and a moderation analysis were planned with holistic school belonging as the dependent variable. However, given concerns over possible multidimensionality in the PSSM, an exploratory factor analysis (EFA) was conducted. The EFA revealed that a holistic measure of school belonging was not appropriate given its multidimensional nature. An EFA was used rather than a confirmatory factor analysis because prior studies have found varying numbers of factors when using the full measure, and it was not clear how many factors would be present when using the shortened version. Given the two factors observed in the shortened version, we conducted hierarchical regression and moderation analyses for each of the two school belonging factors identified through the EFA.

Hierarchical multiple regressions were conducted to analyze correlates of school belonging among Swedish youth. For regression analyses, three control variables (age, gender and material access) were added in step 1 and eight independent variables (quantity and quality of friendships, perceived ethnic discrimination, and the five SDQ subscales) were added in step 2.

Moderation analyses were then conducted to examine whether these social factors differed between newcomer (*n* = 107) and non-newcomer (*n* = 126) students for both positive and negative perceptions of school belonging. Prior to the analysis, all variables were centred to reduce nonessential multicollinearity. Using the PROCESS macro [97], positive and negative perceptions of school belonging were each regressed on the eight social factors in turn, with newcomer status included as a moderator and age, gender, and material access included as covariates. The interaction term for each analysis was examined.

### Sample characteristics

The sample of 233 youth (129 females) had a mean age of 14.7, *SD* = 1.5, and good access to material necessities, as indicated by the high average material access score, *M* = 3.6 out of 4, *SD* = 0.6 (see Table 1). Newcomers made up 46% of the sample (*n* = 107). Among newcomers, the majority reported having been born in Somalia (29%), Syria (20%), Eritrea (11.2%) or did not respond (13%). The most common languages used to complete the measures were Swedish (67%), Amharic (12%), Arabic (9%), English (5%) and Somali (5%). There was no significant difference between the newcomers and non-newcomer student groups in gender composition (*p* > .05) but newcomers did tend to be older (*M* = 15.4) than non-newcomer students (*M* = 14.2, *p* < .01) and have poorer material access (*M* = 3.4) than non-newcomer students (*M* = 3.8, *p* < .01).

**Table 1:** Summary of psychometric properties and intercorrelations for continuous variables.

Measure	*M*	*SD*	*Range*	*α*	*1*	*2*	*3*	*4*	*5*	*6*	*7*	*8*	*9*	*10*
1. School Belonging	30.99	4.90	15–40	.600	-									
2. Age	14.74	1.49	12–19	-	-.108	-								
3. Material Access	3.63	0.62	1–4	.855	.288**	-.207**	-							
4. Total Friends	30.02	17.78	0–60	-	.087	-.276**	.136*	-						
5. Mean Support from Friends	2.54	0.62	1–4	.801	-.082	.039	-.073	-.196**	-					
6. Mean Perceived Ethnic Discrimination	1.43	0.46	1–3.5	.789	-.494**	-.016	-.142*	.063	.177**	-				
7. Prosocial Behaviour	8.03	1.75	2–10	.559	.263**	.082	.056	.097	-0.061	-0.059	-			
8. Emotional Symptoms	2.71	2.33	0–10	.726	-.381**	-.029	-.209**	-.107	.240**	.415**	-.049	-		
9. Conduct Problems	1.78	1.53	0–8	.452	-.285**	-.011	-.169**	.001	0.094	.399**	-.318**	.445**	-	
10. Hyperactivity	3.80	1.79	1–8	.369	-.246**	-.143*	.024	.114	0.111	.429**	-.061	.398**	.391**	-
11. Peer Problems	2.43	1.54	0–8	.304	-.279**	.060	-.320**	-.148*	0.128	.160*	-.220**	.310**	.228**	.092

*Note*. *n* = 233

**p ≤ .001

*p < .05.

## Results

### Exploratory factor analysis

An exploratory factor analysis was conducted to examine the psychometric properties of our shortened PSSM measure. There was no multicollinearity detected among the nine PSSM survey items so we retained all items (*r*s < .452). We conducted a principal components analysis followed by an oblimin rotation method to extract latent factors (see Table 2). An oblimin rotation allows for correlations between underlying factors and has been used successfully in other factor analyses of the PSSM [81, 82]. A satisfactory KMO, .676, and Bartlett’s Test of Sphericity, χ2(36) = 316.89, p < .001, were observed. Three factors were extracted with eigenvalues above 1 (and percent variance explained): 2.25 (25.05%), 1.92 (21.30%), 1.03 (11.48%). All items loaded onto a factor with a value above .40. The model has an α of .600 and an average inter-item correlation of .148 which is outside the optimal range of .15 - .50 suggested by Clark & Watson [98].

**Table 2:** Factor structure matrix of the three-factor model.

Item	Description	Factor 1	Factor 2	Factor 3
PSSM1	I feel like a real part of my school	**0.582**	0.022	**-0.532**
PSSM3	It is hard for people like me to be accepted here (RC)	0.069	**0.752**	0.067
PSSM6	Sometimes I feel as if I don’t belong here (RC)	-0.047	**0.746**	-0.156
PSSM10	I am included in lots of activities at my school	**0.814**	-0.097	0.023
PSSM11	I am treated with as much respect as other students.	**0.707**	0.128	-0.307
PSSM12	I feel very different from most other students (RC)	0.001	**0.722**	-0.030
PSSM13	I can really be myself at this school, I don’t have to pretend	0.089	-0.037	**-0.743**
PSSM16	I wish I were at a different school (RC)	-0.215	**0.551**	**-0.433**
PSSM17	I feel proud of belonging to my school.	**0.465**	0.146	**-0.726**

*Note*: PSSM = Psychological Sense of School Membership; RC = reverse-coded; factor loadings > 0.40 are bolded.

Based on previous theoretical analysis, we identified that Factor 1 was associated with positive perceptions and school identification and contained only positively worded items, while Factor 2 was associated with negative perceptions and rejection and contained all the negatively worded items. Factor 3 only included one item that loaded uniquely onto the factor and was not clearly associated with previously known or identified dimension. We noted that the uniquely loaded item (PSSM Item 13: “I can really be myself at this school, I don’t have to pretend.”) contains the culturally-specific notion of “being oneself” that may have been misunderstood by our participants. We therefore removed the item and conducted a second EFA using principal component analysis and an oblimin rotation on the remaining eight items. A satisfactory KMO, .660, and Bartlett’s Test of Sphericity, χ^2^(28) = 286.85, *p* < .001, were once again observed for the second EFA (see Table 3). This analysis revealed a two-factor structure in which two factors had an eigenvalue above 1 (percent variance explained): 2.15 (26.84%) and 1.87 (23.44%). Factor 1 contained all the positively worded items and was still associated with *positive perceptions* of school belonging and Factor 2 contained all the negatively worded items and was still associated with *negative perceptions* of school belonging. All items loaded onto one factor with a value above .60.

**Table 3:** Factor structure matrix of the two-factor model.

Item	Description	Factor 1	Factor 2
PSSM1	I feel like a real part of my school	.**742**	.040
PSSM3	It is hard for people like me to be accepted here (RC)	.020	.**712**
PSSM6	Sometimes I feel as if I don’t belong here (RC)	.037	.**749**
PSSM10	I am included in lots of activities at my school	.**639**	-.173
PSSM11	I am treated with as much respect as other students.	.**706**	.099
PSSM12	I feel very different from most other students (RC)	.014	.**703**
PSSM16	I wish I were at a different school (RC)	.079	.**614**
PSSM17	I feel proud of belonging to my school.	.**742**	.198

*Note*: PSSM = Psychological Sense of School Membership; RC = reverse-coded; factor loadings > 0.40 are bolded.

Both factors were further investigated using Cronbach’s alpha and average inter-item correlations between and within the two factors. The *positive perceptions* factor had an α of .662 and an average inter-item correlation of .335, while the *negative perceptions* factor had an α of .664 and an average inter-item correlation of .319. The two factors were not significantly correlated with one another, r = .059, p = .373, suggesting that they truly measure separate dimensions.

### The relations between social factors and positive perceptions of school belonging

The regression analysis (see Table 4) revealed that at step 1, a model with control variables age, gender, and material access was significant and accounted for 4% of variance in school belonging. Material access was significantly and positively associated with school belonging, indicating greater material access was associated with higher positive perceptions of school belonging, but age and gender were not.

**Table 4:** Summary of multivariate regression models for correlates with positive and negative school belonging.

		Positive School Belonging					Negative School Belonging				
Independent Variables		*B*	*SE*	β	*t*	*p*	*B*	*SE*	β	*t*	*p*
Step 1	Age	0.11	0.16	0.04	0.66	.509	-0.44	0.15	-0.19	-2.89	**.004**
	Material Access	1.14	0.39	0.20	2.95	**.003**	0.87	0.36	0.15	2.37	**.018**
	Gender (Girl)*	-0.45	0.47	-0.06	-0.96	.340	0.28	0.44	0.04	0.64	.523
		*R*^2^ = .040, adj. *R*^2^ = .027, *F*(3,229) = 3.177, *p* = .025					*R*^2^ = .074, adj. *R*^2^ = .061, *F*(3,229) = 6.06, *p* < .001				
Step 2	Age	-0.102	0.15	-0.04	-0.67	.501	-0.48	0.14	-0.21	-3.40	.**001**
	Material Access	0.686	0.37	0.12	1.85	.066	0.24	0.35	0.04	0.69	.488
	Gender (Girl)*	-0.37	0.45	-0.05	-0.82	.415	0.27	0.43	0.04	0.63	.528
	Total Friends	-0.01	0.01	-0.07	-1.09	.276	0.01	0.01	0.03	0.56	.579
	Support from Friends	0.27	0.36	0.05	0.77	.445	0.53	0.34	0.09	1.57	.117
	Perceived Ethnic Discrimination	-1.14	0.54	-0.15	-2.10	**.037**	-3.28	0.51	-0.44	-6.41	**< .001**
	Prosocial Behaviour	0.69	0.13	0.33	5.15	**< .001**	0.06	0.13	0.03	0.47	.636
	Emotional Symptoms	-0.42	0.12	-0.27	-3.54	**< .001**	-0.04	0.11	-0.03	-0.37	.711
	Conduct Problems	0.15	0.17	0.06	0.85	.399	0.14	0.16	0.06	0.86	.391
	Hyperactivity	-0.14	0.14	-0.07	-1.01	.314	-0.03	0.13	-0.02	-0.25	.800
	Peer Problems	0.09	0.15	0.04	0.56	.575	-0.36	0.14	-0.16	-2.46	**.015**
		*R*^2^ = .261, adj. *R*^2^ = .224, *F*(11,221) = 7.095, *p* < .001					*R*^2^ = .289, adj. *R*^2^ = .254, *F*(11,221) = 8.17, *p* < .001				

*Note*. *n* = 233

*Male reference group.

Introducing the variables of interest in step 2 explained an additional 22.1% of variance in school belonging and this change in *R*^2^ was significant. With all 11 variables included in the model, prosocial behavior was the strongest correlate (β = 0.33), followed by emotional symptoms (β = -0.27) and perceived ethnic discrimination (β = -0.15). Higher school belonging on the positive perceptions dimension was associated with lower perceived ethnic discrimination, lower emotional symptoms, and higher prosocial behaviour. Material access became a marginal control variable. There was no significant association between school belonging and age, gender, total friends, support from friends, conduct problems, or peer problems.

### The relations between social factors and negative perceptions of school belonging

The regression analysis (see Table 4) revealed that at step 1, a model with control variables was significant, and accounted for 7.4% of variance in negative perceptions. Material access was significantly associated with school belonging, as was age. Greater access to material goods and younger age were associated with higher school belonging.

Introducing the variables of interest in step 2 explained an additional 21.5% of variance in school belonging and this change in *R*^2^ was significant. With all 11 variables included in the model, age remained a significant control variable and two social factors were significant: perceived ethnic discrimination and peer problems. Perceived ethnic discrimination was the greatest correlate (β = -0.44), followed by age (β = -0.21) and peer problems (β = -0.16).

### Exploring newcomer status as a potential moderator

The interaction term for each analysis was examined to assess whether moderation occurred. Table 5 summarizes the interaction terms for all analyses.

**Table 5:** Summary of interaction terms from moderation analyses for social correlates with positive and negative school belonging.

	*Positive Perceptions of School Belonging*				*Negative Perceptions of School Belonging*			
Interaction Term	*b*	*SE*	*t*	*p*	*b*	*SE*	*t*	*p*
	[95% CI]				[95% CI]			
Newcomer Status x Total Friends	-0.0075 [-0.02, 0.01]	0.01	-0.96	.336	0.01 [-0.01, 0.02]	0.01	0.86	.388
Newcomer Status x Support from Friends	-.0151 [-0.28, 0.25]	0.13	-0.11	.910	-0.12 [-0.38, 0.14]	0.13	-0.92	.360
Newcomer Status x Perceived Ethnic Discrimination	0.03 [-0.22, 0.28]	0.13	0.24	.808	-0.03 [-0.26, 0.20]	0.11	-0.26	.792
Newcomer Status x Prosociality	0.16 [-0.08, 0.41]	0.12	1.32	.187	-0.02 [-0.27, 0.23]	0.13	-0.16	.869
Newcomer Status x Emotional Problems	-0.27 [-0.52, -0.03]	0.12	-2.21	.**028**	-0.08 [-0.32, 0.17]	0.13	-0.61	.541
Newcomer Status x Conduct Problems	-0.10 [-0.36, 0.15]	0.13	-0.80	.425	0.06 [-0.19, 0.31]	0.13	0.46	.643
Newcomer Status x Hyperactivity	-0.12 [-0.39, 0.15]	0.14	-0.86	.389	-0.10 [-0.37, 0.17]	0.14	-0.72	.469
Newcomer Status x Peer Problems	-0.20 [-0.46, 0.06]	0.13	-1.52	.129	0.09 [-0.16, 0.35]	0.13	0.73	.463

*Note*. *n* = 233.

For positive perceptions of school belonging, only one significant interaction was found reflecting a moderation effect of newcomer status on the relationship between emotional symptoms and positive school belonging, *p* = .028. No other interactions were significant, *p*s > .100. The significant interaction reflects a steeper negative slope between emotional symptoms and positive perceptions of school belonging for newcomers than non-newcomers.

The same set of analyses were run with negative perceptions as the dependent variable. No significant interactions were found, *p*s > .300, suggesting that newcomer status does not moderate the relationship between negative school belonging and any of the social factors when controlling for age, gender, and material stress.

## Discussion

Our study set out to (1) understand which social factors were associated with school belonging in a diverse sample of youth in Sweden, and (2) investigate whether newcomer status moderates the relationship between social variables and youths’ sense of school belonging.

### Social factors associated with school belonging

Our analyses revealed that perceived ethnic discrimination is an important correlate of school belonging, regardless of whether the positive or negative perceptions of school belonging are considered. For both dimensions, a stronger sense of school belonging was associated with perceiving less ethnic discrimination. This is consistent with other research showing the deleterious effects of discrimination on feelings of belonging [44, 46] and raises concerns due to reports of high levels of discrimination within schools, especially for newcomers [66, 99–102]. For example, Somali youth in Sweden have previously reported everyday racism in their lives as a considerable stressor, not least at school [66].

With regard to positive perceptions of school belonging, the other significant associations were prosocial behaviour and emotional symptoms. These findings are consistent with the literature that finds lower school belonging among those with emotional problems like depression [23, 33, 39], and higher school belonging among students with higher “positive personal characteristics” like prosociality [17, 25, 33, 51, 52]. Notably, prosocial behaviour was the most important correlate of positive perceptions of school belonging, suggesting it may be an ideal target for asset-building interventions.

However, we are aware that some items on the SDQ’s prosociality scale may measure social conformity. If so, our results instead suggest that youth who are more likely to *conform to social norms* express a stronger sense of school belonging. This association is unsurprising: exclusion or a loss of social status is a known risk for youth who deviate from social norms [103] and adolescents are especially sensitive to such risks [104]. This alternative interpretation, if true, would suggest that interventions to increase inclusivity and tolerance for violated social norms may be beneficial to supporting school belonging.

Material access was also a marginally significant control variable, consistent with prior research showing higher school belonging among more economically advantaged students [18,64].

In regard to negative perceptions, an additional two variables were significant: age and peer problems. This is consistent with large-scale research showing youths’ school identification decreases across the teenage years [40, 50, 61] and the literature reporting the importance of positive peer relationships for establishing a sense of school belonging [37, 38, 40–42]. Notably, personal factors like problematic peer relationships are associated with factors outside the individual such as public policy decisions like classroom integration procedures or funding for social-emotional skills building [105]. Therefore, interventions aimed at reducing peer problems as a means of building school belonging should consider the use of both structural changes and individual-level actions.

Interestingly, four social variables were not associated with any dimension of school belonging: quality and quantity of peer relationships, conduct problems, and hyperactivity. The absence of quality of peer relationships is perhaps the most surprising finding because relationships with peers are often highlighted as an important element of feeling belonging at school. One possible explanation for our findings is that students may have struggled to respond to the measure of peer relationship quality, resulting in more error variance and non-significant findings. For example, youth may have struggled to report on the average support from friends if they have many groups of friends who supported them to differing degrees. This interpretation may be supported by the weak or non-existent correlations between quality of peer relationships and the related variables of prosociality and peer problems (see Table 1), and by number of friendships being the most common item of missing data. Another possibility is that peer relationship quality may not be as important in establishing school belonging as previously thought. This is not consistent with most prior literature. However, there is a body of work demonstrating that relationships with teachers tend to be more important for school belonging than peer relationships [38, 40, 106].

Quantity of peer relationships may not have been a significant correlate if there is a non-linear relation between youths’ number of friendships youth and sense of school belonging; youth may simply need to reach a threshold of friendships in order to experience a sense of school belonging, or they may receive a diminishing benefit from more peer relationships. This would be consistent with findings of Ren and colleagues [107] who have observed that one’s quantity of social contact provides “diminishing returns” to wellbeing.

### Newcomer status as a moderator

Our second research aim focussed on newcomer status as a potential moderator. We aimed to investigate whether some social variables may predict school belonging differently for newcomers based on their unique life experiences and social circumstances. We found instead that the social variables we examined were more universally related to school belonging and that there was no consistent evidence of newcomer status moderating the relationship. In our analysis, we did control for material access—a marker of economic situation. Newcomers tend to be more economically disadvantaged, as was the case for our sample. We therefore conducted a sensitivity analysis to determine if newcomer status would emerge as a moderator had we not controlled for material access. We found no consistent evidence for this and noted the same pattern of results as when material access was included as a covariate.

### Strengths and limitations

Our study addressed an important gap in the literature using data from a diverse sample of youth recruited from different schools. The diversity in ethnicity, geography, language background, and newcomer status of our sample adds to the generalizability of our results. We also examined a variety of social variables which was beneficial in revealing which social variables may be the strongest correlates of school belonging and therefore the most relevant targets for asset-building interventions or policy reviews. Furthermore, we used measures with strong psychometric properties that support the validity and reliability of our findings.

However, our study was also limited in many ways. First, we did not use the full PSSM scale due to time constraints. We excluded PSSM items related to connections with teachers which tend to reflect an additional dimension of school belonging [81, 82] and may be predicted by different variables. We also made the survey available in many languages to accommodate the diverse needs within our sample. While this allowed us to recruit a more diverse sample and increased our confidence that youth understood the survey items, it also may have introduced additional error variance into the sample as youth may have interpreted items slightly differently based on the language in which they completed the survey. Additionally, while we successfully identified significant models that explain a substantial amount of variance (26.1% and 28.9% respectively), there is still much unexplained variance. Other elements of school belonging outside of social factors, including structural factors, are likely important as well.

We also noted a low participation rate, with only 42% of invited youth providing complete data. While this response rate is within the typical range of 40–50% participation in health surveys [108], it can still create bias if some groups are more likely to participate than others.

Furthermore, we observed poor internal consistency for several SDQ subscales including conduct problems, hyperactivity, and peer problems. The SDQ is a widely used measure so its inclusion, in full and without alteration, provides many benefits for comparison to other samples. The measure has also been shown to have other strong psychometric properties, such as good specificity and negative predictive value [e.g., 109] and strong convergent and divergent validity [e.g., 110], which supports its inclusion in this study. However, given the low internal consistency we observed on some subscales, conclusions related to these subscales should be interpreted with caution.

A final limitation of the work is that we cannot provide a definitive interpretation of the negative school belonging factor. Like others, we found the PSSM to be multidimensional with positive and negatively worded items loading only with similar items. The factor containing negatively worded items has been interpreted by some to represent rejection [77, 79] and similar negatively worded items have been described as “measuring a sense of belonging by assessing a lack of not belonging” [111; p. 311]. However, others have argued that the factor reflects a method effect created by the items’ wording [81, 82]. In our work, we use the term *negative perceptions of school belonging* because it is unclear whether this factor represents rejection, a method effect, or some combination of the two. Therefore, it is not entirely clear if the factors associated with negative perceptions of school belonging in our study (age, perceived ethnic discrimination, and peer problems) are associated with levels of rejection, more sensitivity to negatively worded items, or both.

### Future directions

This work raises several exciting future directions for research. First, the directionality of the significant associations found warrant further investigation. Understanding whether prosocial and emotionally problematic behaviours arise from one’s sense of school belonging, cause that sense of school belonging, or both, is important for interventionists seeking to boost school belonging. Related prior literature in this area has been mixed. Longitudinal studies to examine the direction of these associations, especially among diverse populations, is clearly needed.

Second, this study contributes to the growing body of literature supporting the multidimensional nature of the PSSM. Future research on the correlates of school belonging must guard against treating the PSSM as a unidimensional measure, or else use newer unidimensional tools for measuring school belonging like the Simple School Belonging Scale [112].

Third, while we examined peer relationships and social strengths and difficulties as correlates of school belonging, it is clear from prior literature that other areas of a youth’s life such as the physical environment of the school, the presence of extra-curricular activities and recreation resources, and the degree of academic support received can play a role in school belonging [12, 33]. Other factors less proximal to the individual, such as educational policies and broader cultural beliefs, likely also play a role in school belonging whether by influencing the individual directly or features of their environment like the school’s environment. All these potential correlates also deserve further examination in future work and including them in future models would likely explain even greater variance in school belonging.

Finally, qualitative research would add depth to our understanding of the associations between social factors and youths’ sense of school belonging. Collecting qualitative data related to school belonging would allow for a more fulsome and nuanced examination of the interplay between the social variables and school belonging. This additional depth may also provide answers to remaining questions in the data, such as why quantity and quality of peer relationships did not predict school belonging.

## Conclusions and implications

This study provides evidence that many social factors are associated with Swedish youths’ sense of school belonging and that some of the most important may be perceived ethnic disrimination and prosocial behaviour, strengthening previous findings that school environment and positive interpersonal skills contribute to school belonging. It also provided additional evidence that the PSSM is not a unidimensional measure but has at least two distinct factors: one positive and one negative dimension. Furthermore, the study revealed no consistent evidence for newcomer status moderating the relationship between social variables and school belonging, supporting the conclusion that newcomer students’ school belonging is not associated to their status as such, but to modifiable factors, such as ethnic discrimination, prosocial behaviour, emotional problems, and peer problems.

Our analysis of possible moderation suggests that interventions promoting school belonging do not need to target different sets of social skills for newcomer and non-newcomer students. However, as material deprivation was higher among newcomer students, interventions that improve deprivation may have greater impacts for newcomers.
